# Supplementary material for: PfApiAT2 is a proline transporter essential for the transmission of Plasmodium falciparum by the mosquito vector
Source: Nat Commun. 2026 Jul 3;17:9388. doi: 10.1038/s41467-026-75126-1 (PMC13534427; doi:10.1038/s41467-026-75126-1)
Supplement: Supplementary file 2 [file 41467_2026_75126_MOESM2_ESM.pdf]

Description of additional supplementary files

Supplementary Data 1:

The p-value for the relevant comparisons has been specified in the figure legends, and all comparisons done in the study.
