# Supplementary material for: PfApiAT2 is a proline transporter essential for the transmission of Plasmodium falciparum by the mosquito vector
Source: Nat Commun. 2026 Jul 3;17:9388. doi: 10.1038/s41467-026-75126-1 (PMC13534427; doi:10.1038/s41467-026-75126-1)
Supplement: Supplementary file 3 [file 41467_2026_75126_MOESM4_ESM.pdf]

nature portfolio

Corresponding author(s): Dyann Wirth, Flaminia Catteruccia, Selina Bopp

Last updated by author(s): Jun 5, 2026

Reporting Summary

Nature Portfolio wishes to improve the reproducibility of the work that we publish. This form provides structure for consistency and transparency in reporting. For further information on Nature Portfolio policies, see our Editorial Policies and the Editorial Policy Checklist.

Statistics

For all statistical analyses, confirm that the following items are present in the figure legend, table legend, main text, or Methods section.

n/a	Confirmed	
☐	☒	The exact sample size (*n*) for each experimental group/condition, given as a discrete number and unit of measurement
☐	☒	A statement on whether measurements were taken from distinct samples or whether the same sample was measured repeatedly
☐	☒	The statistical test(s) used AND whether they are one- or two-sided<br>*Only common tests should be described solely by name; describe more complex techniques in the Methods section.*
☐	☒	A description of all covariates tested
☐	☒	A description of any assumptions or corrections, such as tests of normality and adjustment for multiple comparisons
☐	☒	A full description of the statistical parameters including central tendency (e.g. means) or other basic estimates (e.g. regression coefficient) AND variation (e.g. standard deviation) or associated estimates of uncertainty (e.g. confidence intervals)
☐	☒	For null hypothesis testing, the test statistic (e.g. *F*, *t*, *r*) with confidence intervals, effect sizes, degrees of freedom and *P* value noted<br>*Give P values as exact values whenever suitable.*
☒	☐	For Bayesian analysis, information on the choice of priors and Markov chain Monte Carlo settings
☒	☐	For hierarchical and complex designs, identification of the appropriate level for tests and full reporting of outcomes
☒	☐	Estimates of effect sizes (e.g. Cohen's *d*, Pearson's *r*), indicating how they were calculated

Our web collection on statistics for biologists contains articles on many of the points above.

Software and code

Policy information about availability of computer code

Data collection	No software was used for data collection
Data analysis	GraphPad Prism 10.0, FiJi ImageJ version 2.9.0/1.53t, 3D7 reference genome from PlasmoDB version 13, Pymol 2.5.2, RStudio version 2024.12.1+563, OocystMeter (https://www.biorxiv.org/content/10.1101/2025.06.28.662088v1), ZEN version 3.0.

For manuscripts utilizing custom algorithms or software that are central to the research but not yet described in published literature, software must be made available to editors and reviewers. We strongly encourage code deposition in a community repository (e.g. GitHub). See the Nature Portfolio guidelines for submitting code & software for further information.

Data

Policy information about availability of data

All manuscripts must include a data availability statement. This statement should provide the following information, where applicable:

- Accession codes, unique identifiers, or web links for publicly available datasets
- A description of any restrictions on data availability
- For clinical datasets or third party data, please ensure that the statement adheres to our policy

All other data supporting the findings are available in the Supplementary Information and in the Source Data file.

Research involving human participants, their data, or biological material

Policy information about studies with human participants or human data. See also policy information about sex, gender (identity/presentation), and sexual orientation and race, ethnicity and racism.

Reporting on sex and gender	Human red blood cells (RBCs) and serum were used for the Plasmodium falciparum culture. Both were sourced from anonymous healthy male donors.
Reporting on race, ethnicity, or other socially relevant groupings	RBCs and serum sourced from O+ve white male donors from Research Blood Components (Watertown, MA, USA) and Interstate Blood Bank (Memphis, TN, USA). All donors were anonymous
Population characteristics	All additional donor characteristics were anonymized prior to receiving RBCs and serum and are unknown to the researchers.
Recruitment	No specific recruitment was conducted. Anonymous donors gave blood and serum at above locations
Ethics oversight	All experiments conducted following relevant NIH guidelines and regulations. Samples were sourced by outside vendors in accordance with relevant informed consent policies.

Note that full information on the approval of the study protocol must also be provided in the manuscript.

Field-specific reporting

Please select the one below that is the best fit for your research. If you are not sure, read the appropriate sections before making your selection.

☒ Life sciences ☐ Behavioural & social sciences ☐ Ecological, evolutionary & environmental sciences

For a reference copy of the document with all sections, see nature.com/documents/nr-reporting-summary-flat.pdf

Life sciences study design

All studies must disclose on these points even when the disclosure is negative.

Sample size	Parasite growth assays and mosquito infection experiments were all performed in triplicate, unless specified, as is the norm in the field (Probst et al. 2025 Nature, PMID: 40399670). Researchers aimed for a minimum of >16 mosquitoes per group per replicate, and on rare occasion fewer were dissected due to poor mosquito mortality or feeding. For immunofluorescence assay, all parasites per midgut per replicate were assessed. For oocyte flux assays, 7-10 oocytes per group per replicate were assessed in a minimum of 4 biological replicates, as is the norm in the field (Parker, et al. 2019 PLos Pathog., PMID: 34432856).
Data exclusions	No data was excluded.
Replication	Parasite growth experiments were performed in triplicate. For mosquito infection experiments, three to four replicates were performed, all of which were successful. For Xenopus oocyte flux experiments, four to five biological replicates were performed, all of which were successful.
Randomization	Mosquitoes were collected as pupae and randomly assigned to different cages representing distinct infection and treatment groups. For oocyst and sporozoite quantification, mosquitoes were either randomly selected, or all remaining mosquitoes (if terminal timepoint) were collected. For Xenopus flux uptake experiments, healthy oocytes were randomly assigned to tubes representing different flux conditions.
Blinding	Blinding was not necessary as all mosquito infections were analyzed using the software OocystMeter (bioRxiv Peng, 2025). Blinding was not necessary for immunofluorescence assays as only one genotype was assessed. Researchers were not blinded for quantitative readouts of Xenopus radio-flux.

Reporting for specific materials, systems and methods

We require information from authors about some types of materials, experimental systems and methods used in many studies. Here, indicate whether each material, system or method listed is relevant to your study. If you are not sure if a list item applies to your research, read the appropriate section before selecting a response.

Materials & experimental systems

n/a	Involved in the study
☐	☒ Antibodies
☐	☒ Eukaryotic cell lines
☒	☐ Palaeontology and archaeology
☐	☒ Animals and other organisms
☒	☐ Clinical data
☒	☐ Dual use research of concern
☒	☐ Plants

Methods

n/a	Involved in the study
☒	☐ ChIP-seq
☒	☐ Flow cytometry
☒	☐ MRI-based neuroimaging

Antibodies

Antibodies used	Anti HA host Rabbit Source: RM305, SAB5600116, Sigma-Aldrich;Anti HA host Mouse Source: 16B12, Sigma-Aldrich;Anti GAPDH host Mouse Source: MABS1946, EMD Millipore;Anti Cap380 host Rabbit Source: This study.
Validation	Anti-HA antibodies were validated by staining WT and HA-tagged samples. Anti-GAPDH antibody (MABS1946, EMD Millipore) evaluated by Western Blotting in Plasmodium falciparum infected human erythrocytes. Anti-Cap380 antibody validated on P. falciparum infected mosquito midguts.

Eukaryotic cell lines

Policy information about cell lines and Sex and Gender in Research

Cell line source(s)	NF54 P. falciparum cell line from BEI Resources (MRA-1000)
Authentication	Parasite line was authenticated using nested PCR protocol with primers specific to P. falciparum. WT and mutant parasites verified by Sanger sequencing. WT and transgenic parasites validated via PCR around integration sites
Mycoplasma contamination	All P. falciparum strains were confirmed to be free of mycoplasma contamination.
Commonly misidentified lines (See ICLAC register)	*Name any commonly misidentified cell lines used in the study and provide a rationale for their use.*

Animals and other research organisms

Policy information about studies involving animals; ARRIVE guidelines recommended for reporting animal research, and Sex and Gender in Research

Laboratory animals	Anopheles gambiae (G3 strain) female mosquitoes aged 4-7 days post-eclosion at the onset of experiment. Adult female Xenopus laevis frogs were used for oocytes.
Wild animals	No wild animals were used for this study
Reporting on sex	Only female mosquitoes used, as only female mosquitoes feed on blood. Only female Xenopus frogs were used, as only female frogs produce oocytes.
Field-collected samples	No field collected samples were used in the study.
Ethics oversight	Research involving non-human vertebrates (Xenopus laevis) was conducted under approval and oversight of the Northeastern University Institutional Animal Care and Use Committee (NU-IACUC), Protocol #25-0924R (‘Heterologous Expression Studies in Xenopus laevis Oocytes’; approval date September 29, 2025; expiration September 1, 2028). Northeastern University’s animal care and use program is AAALAC International accredited and holds OLAW Assurance number D16-00095. Lower invertebrates, such as mosquitoes, are exempt from regulations governing the use of animals in research.

Note that full information on the approval of the study protocol must also be provided in the manuscript.

Plants

Seed stocks	N/A
Novel plant genotypes	N/A
Authentication	N/A
