# Supplementary material for: PfApiAT2 is a proline transporter essential for the transmission of Plasmodium falciparum by the mosquito vector
Source: Nat Commun. 2026 Jul 3;17:9388. doi: 10.1038/s41467-026-75126-1 (PMC13534427; doi:10.1038/s41467-026-75126-1)
Supplement: Supplementary file 4 [file 41467_2026_75126_MOESM5_ESM.pdf]

nature portfolio

Peer Review File

PfApiAT2 is a proline transporter essential for the transmission of *Plasmodium falciparum* by the mosquito vector.

Corresponding Author: Professor Dyann Wirth

This file contains all reviewer reports in order by version, followed by all author rebuttals in order by version.

Version 0:

Reviewer comments:

Reviewer #1

(Remarks to the Author)
This m/s by Khushu and colleagues demonstrates a key role of the Plasmodium proline transporter ApiAT2 in Plasmodium falciparum oocyst development and transmission. The work is elegantly conducted and well-presented and the data are robust and convincing. My compliments to the authors on this nice piece of research!

I have just a few minor comments:

I am surprised that the authors did not try to rescue the ApiAT2-KO phenotype in mosquito with a dietary supplement of just proline rather than the amino acid mixture. I am not asking this experiment be included, as the evidence of ApiAT2 as a proline transporter is solid, but I am curious if there was a particular reason for not doing this?

Line 236/7: Please cite reference Saeed et al., 2023 (PMID38097730) which supports their statement that the capsule is permeable to small molecules.

Reference 28: Please use or add a more recent review on crystalloids, Dessens et al 2021 (PMID33941493).

Reviewer #2

(Remarks to the Author)
This is a fairly robust study that identifies PfApiAT2 as a proline-specific transporter essential for P falciparum oocyst development in Anopheles mosquitoes. While dispensable during asexual blood stages, its loss by drug-selection mutations or genetic ablation, results in stunted oocysts that fail to produce sporozoites. Interestingly, this defect is rescued by supplementing the mosquito with exogenous nutrients. The ApiAT2 is shown to be implicated. The findings are significant with potential translational implications.

Shortcomings are:
A previous study has shown that P. beghei ApiAT2 is essential for sporogonic development; the discovery of a similar (same) function in P. falciparum is hence not entirely novel. The mechanistic insights are however novel.

The question of whether stunted oocysts are dead or permanently stalled could be addressed experimentally with dyes.

The hint at oocysts are capable of synthesizing proline from other amino acids could be addressed experimentally since that has been shown for other parasites. Knocking out arginine biosynthetic pathway in ApiAT2-KO parasites and then test rescuing with additional blood meals would better confirm mechanism. RNAseq assays may also provide insights.

It is proposed that drugs in the bednet inhibiting the transporter could block the parasite and thereby transmission. The question is whether drug that stalls oocyst development would continue to act for the required duration to completely abolish

sporozoite development.

Version 1:

Reviewer comments:

Reviewer #1

(Remarks to the Author)
I am happy with the response.

Reviewer #2

(Remarks to the Author)
The authors have constructively addressed reviewers comments with appropriate editing of the manuscript and addition of data.

Open Access This Peer Review File is licensed under a Creative Commons Attribution 4.0 International License, which permits use, sharing, adaptation, distribution and reproduction in any medium or format, as long as you give appropriate credit to the original author(s) and the source, provide a link to the Creative Commons license, and indicate if changes were made.
In cases where reviewers are anonymous, credit should be given to 'Anonymous Referee' and the source.
The images or other third party material in this Peer Review File are included in the article's Creative Commons license, unless indicated otherwise in a credit line to the material. If material is not included in the article's Creative Commons license and your intended use is not permitted by statutory regulation or exceeds the permitted use, you will need to obtain permission directly from the copyright holder.
To view a copy of this license, visit https://creativecommons.org/licenses/by/4.0/

Dear reviewers,

Thank you for offering your insightful comments to our manuscript “PfApiAT2 is a proline transporter essential for the transmission of *Plasmodium falciparum* by the mosquito vector”. We appreciate the effort taken and have attempted to address these comments in our attached draft. These changes are highlighted as tracked changes, and please find below our response (in blue text) to the provided comments.

Reviewer #1 (Remarks to the Author):

This m/s by Khushu and colleagues demonstrates a key role of the Plasmodium proline transporter ApiAT2 in Plasmodium falciparum oocyst development and transmission. The work is elegantly conducted and well-presented and the data are robust and convincing. My compliments to the authors on this nice piece of research!

Thank you for the kind words for our manuscript!

I have just a few minor comments:

I am surprised that the authors did not try to rescue the ApiAT2-KO phenotype in mosquito with a dietary supplement of just proline rather than the amino acid mixture. I am not asking this experiment be included, as the evidence of ApiAT2 as a proline transporter is solid, but I am curious if there was a particular reason for not doing this?

We have performed this experiment and have now included the results in the manuscript (Supplementary Figure Data 6B, pages 9-10, lines 223-228) and in the discussion (page 12, line 279). We found that individual supplementation of proline (20mM) provided in sugar from the time of infection does not rescue PfApiAT2-KO parasites. This experiment demonstrates that the rescue effect of a mixture of amino acids or a supplemental blood meal cannot readily be replaced by supplementation with a single amino acid, and suggests that PfApiAT2 may be the only proline transporter expressed by growing oocysts.

Line 236/7: Please cite reference Saeed et al., 2023 (PMID38097730) which supports their statement that the capsule is permeable to small molecules.

This change has been made on page 10, lines 246-247 in the revised manuscript, thank you for pointing this out (Citation #33).

Reference 28: Please use or add a more recent review on crystalloids, Dessens et al 2021 (PMID33941493).

This change has been made on page 6, line 146 in the revised manuscript, thank you for pointing this out (Citation #30).

Reviewer #2 (Remarks to the Author):

This is a fairly robust study that identifies PfApiAT2 as a proline-specific transporter essential for P falciparum oocyst development in Anopheles mosquitoes. While dispensable during asexual blood stages, its loss by drug-selection mutations or genetic ablation, results in stunted oocysts that fail to produce sporozoites. Interestingly, this defect is rescued by supplementing the mosquito with exogenous nutrients. The ApiAT2 is shown to be implicated. The findings are significant with potential translational implications.

Shortcomings are:
A previous study has shown that P. beghei ApiAT2 is essential for sporogonic development; the discovery of a similar (same) function in P. falciparum is hence not entirely novel. The mechanistic insights are however novel.

We thank the reviewer for pointing out the novelty of our mechanistic findings.

The question of whether stunted oocysts are dead or permanently stalled could be addressed experimentally with dyes.

While our study does not precisely determine the timing of oocyst death after stalling, in Figure 6B we describe PfApiAT2-KO oocysts are able to be partially rescued following an additional blood-meal delivered around the time of growth arrest. To answer your question whether a subset of PfApiAT2-KO oocysts does not respond to additional nutrients, we plotted the size (measured at 7 days post infection) of **all** PfApiAT2-KO oocysts from every mosquito from figure 6B (comparing mosquitoes provided a single infectious blood-meal (1BF), a second non-infectious blood-meal (2BF) or a protein-meal (BSA), Response Figure 1).

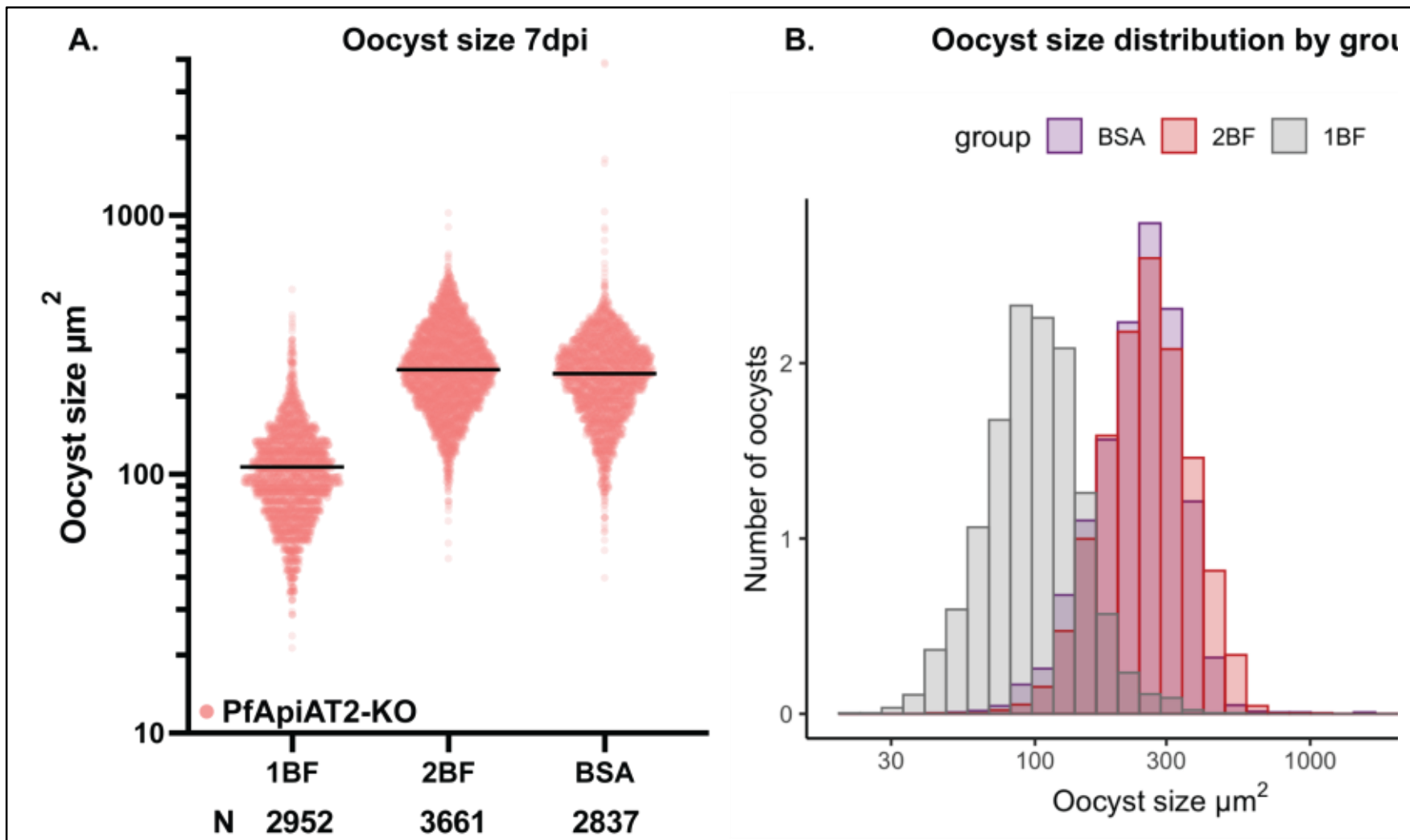

Response Figure 1: Distribution of size of all PfApiAT2-KO oocysts at 7dpi from Figure 6B as either a (A) scatter-plot or (B) overlapping histograms. The distributions of PfApiAT2-KO under both baseline nutrition (1BF) or nutritionally supplemented conditions (2BF and BSA) show only one peak, suggesting that the entire population is responding to additional nutrients, and a subpopulation of non-responsive dead oocysts does not exist.

As you will see from the Response Figure 1, we observed that the overall distributions of oocyst size from the 2BF and BSA groups is increased as compared to the 1BF group, and we do not observe a population of oocysts that remains small in the presence of additional nutrients. We have included this data (Response Figure 1A) as part of the revised manuscript as Supplemental Figure 6A, and have included a description of the results on page 9 lines 207-210. Additional data presented in Figure 4 shows that PfApiAT2-KO oocysts have no ultrastructural signs of damage or stress by TEM at 7dpi. While we cannot rule out that a small number have died at this timepoint, we

believe this suggests that most of the PfApiAT2-KO oocysts are alive but stalled at day 7, and their growth can be rescued with an additional bloodmeal or BSA.

Interestingly, however, in the absence of additional nutrients PfApiAT2-KO oocysts eventually degenerate, as demonstrated by the ultrastructural abnormalities we observed at 14dpi, shown in Figure 4. This suggests that PfApiAT2-KO oocysts accrue ultrastructural abnormalities between 7-14dpi. In this revised manuscript, we have elaborated on our interpretation of the data in the discussion on pages 11-12 lines 272-275 with the following text: “We also observed that PfApiAT2-KO oocysts are morphologically normal up until at least 7dpi but show aberrations by 14dpi, suggesting they can withstand starvation for a few days, likely by entering a stalled state from which they can be rescued by additional nutrients, as has been previously suggested for *P. berghei* [8]”

The hint at oocysts are capable of synthesizing proline from other amino acids could be addressed experimentally since that has been shown for other parasites. Knocking out arginine biosynthetic pathway in ApiAT2-KO parasites and then test rescuing with additional blood meals would better confirm mechanism. RNAseq assays may also provide insights.

We agree with the reviewer that these experiments would help addressing this specific point. However, they would require a substantial technical and temporal effort that we believe goes beyond the scope of this current work, especially given recent evidence from asexual parasites strongly supporting our hypothesis. Indeed, we have demonstrated in our recent publication (Bopp 2025, PMID: 41061700) that the arginine to proline biosynthetic pathway is active in asexual stage parasites and contributes a significant proportion of the parasite cytosolic proline pool. There were two lines of evidence supporting this conclusion: 1) Isotopically labeled arginine can serve as a precursor for proline in asexual parasites; and 2) knockout of the gene encoding ornithine aminotransferase (PfOAT) demonstrated the requirement for arginine to proline conversion in *P. falciparum* blood stage parasites.
Interestingly, recent published work from our group (Yan, Cheung, Vezier, 2024, PMID: 41125888) has used single-cell RNA-seq to detect gene expression in mosquito stages. Transcripts from enzymes in the arginine to proline biosynthetic pathway in oocysts are detected (Response Figure 2), indicating that this pathway is likely active. In the revised manuscript we have expanded our discussion of these recently published results to strengthen our hypothesis with the addition of the following text on page 12 lines 275-280: “In asexual blood-stage parasites, *Plasmodium* can synthesize proline from arginine via ornithine, and this pathway contributes to dysregulation of proline homeostasis in asexual stage PfApiAT2-KO parasites [2, 21, 41]. We hypothesize this is the pathway through which PfApiAT2-KO oocysts are rescued under nutrient supplementation conditions (but not by proline alone), a hypothesis supported by the detection of transcripts of enzymes in this pathway during oocyst development [21, 40]. ”

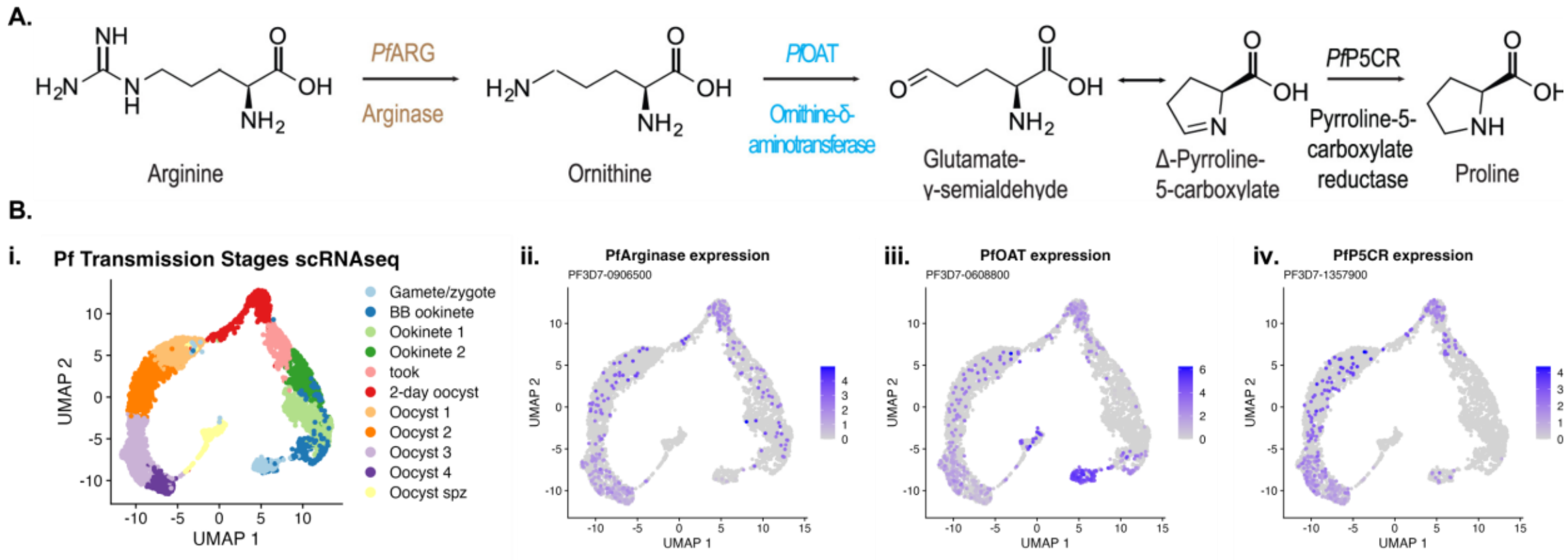

Response Figure 2: The arginine to proline biosynthetic pathway in *P. falciparum* is expressed in transmission stages. (A) The arginine to proline biosynthetic pathway in *P. falciparum* involves three enzymes. *Figure from Bopp 2025, PMID: 41061700*. (B) (i) UMAP of scRNA-seq showing the transmission stages of *P. falciparum*. (ii-iv) Expression of the three enzymes involved in the arginine to proline biosynthetic pathway in these stages. *P. falciparum* expresses the enzymes necessary to convert arginine to proline during oocyst developments. *Data from Yan, Cheung, Verzier, 2024, PMID: 41125888.*

It is proposed that drugs in the bednet inhibiting the transporter could block the parasite and thereby transmission. The question is whether drug that stalls oocyst development would continue to act for the required duration to completely abolish sporozoite development.

We agree that targeting PfApiAT2 may only stall oocyst development and delay (rather than abolish) sporozoite production. However, recent research into bed net-based antimalarials has suggested that delaying sporozoite formation would have a strong effect on overall chance of transmission. Drugs that target metabolic pathways in the parasite can delay oocyst development after oocyst infection is established (Probst, Paton, 2025, PMID: 40399670, Paton, 2022, PMID: 35687594). Even a few days delay in oocyst development (and subsequent sporozoite arrival to the salivary glands) is predicted by mathematical models of malaria transmission to cause a significant decline in vectorial capacity (Kamiya, 2022, PMID: 36209032) given the short lifespan of adult mosquitoes in the field (Costantini, 1996, PMID: 8887330). To accommodate your comment, we have rephrased text on page 13, lines 301-304:

1. Original text: “Our data uncovering proline homeostasis and scavenging as critical for oocyst growth now reveal PfApiAT2 as a promising druggable candidate for transmission-blocking strategies aimed at the mosquito vector, expanding the malaria control toolbox.”
2. Rephrased text: “Our data uncovering proline homeostasis as critical for oocyst growth suggest factors essential for nutrient scavenging by the parasite in the mosquito vector may be promising druggable candidates for transmission-blocking strategies, expanding the malaria control toolbox.”

Yours sincerely,

Selina, Flaminia and Dyann
